# SDMdata: A Web-Based Software Tool for Collecting Species Occurrence Records

**DOI:** 10.1371/journal.pone.0128295

**Published:** 2015-06-01

**Authors:** Xiaoquan Kong, Minyi Huang, Renyan Duan

**Affiliations:** Department of Life Science, Anqing Normal University, Anqing, Anhui, 246011, PR China; J. Craig Venter Institute, UNITED STATES

## Abstract

It is important to easily and efficiently obtain high quality species distribution data for predicting the potential distribution of species using species distribution models (SDMs). There is a need for a powerful software tool to automatically or semi-automatically assist in identifying and correcting errors. Here, we use Python to develop a web-based software tool (SDMdata) to easily collect occurrence data from the Global Biodiversity Information Facility (GBIF) and check species names and the accuracy of coordinates (latitude and longitude). It is an open source software (GNU Affero General Public License/AGPL licensed) allowing anyone to access and manipulate the source code. SDMdata is available online free of charge from <http://www.sdmserialsoftware.org/sdmdata/>.

## Introduction

Species occurrence records are very important for building species distribution models (SDMs) for use in assessing the potential niches of species [1], testing conservation applications, evolutionary and biogeographical hypotheses [2–4], projecting species invasion risk and proliferation [5], assessing the impact of climate change, land use, urbanization and other environmental changes [6–8], suggesting potential suitable sites for rare species [9], and supporting appropriate conservation planning and reserve selection [10].

The majority of species distribution data sets come from databases, museums, herbaria, and field workers [11]. Among these, the Global Biodiversity Information Facility (GBIF; http://www.gbif.rog/) is the largest provider of species distribution records, with a web-based API (Application Programming Interface) to help users efficiently fetch data. GBIF’s API is program-oriented, and several software packages provide functions to help users connect to it. For example, rgbif is a small, open source package linking to GBIF based on R language [12]. However, using R language consumes a large amount of memory, and some users are not familiar with this sometimes idiosyncratic language. Another software providing GBIF API function is modestR. ModestR is a powerful and fully-stacked software to download, import and clean species distribution records [13]. ModestR is a huge software package (the size is more than 4.6GB in the latest version) written in C# language that can only be used in a Windows operating system in the current version, which limits its application.

With the development of species distribution models, studying the effect of environmental change or reserve size on species distribution requires a large number of species distribution data [14,15]. For example, Munguía et al. [14] used the distribution data of 5544 amphibian species to measure how equilibrium varies between species distributions and climate across taxa and regions at a global scale. Cantú-Salazar and Gaston [15] obtained geographical range maps for 1878 terrestrial mammal, 4100 bird and 3096 amphibian species to explore discrepancies between species richness and geographical range maps and test possible effects of reserve size on range maps. In general, one species has 10~10,000 data records, such that significant time is required to obtain and check the accuracy of many records. There is a need for a powerful but easy-to-use, web-based software to meet the requirements of big data. Python is a widely used programming language (https://www.python.org/) that is often used in writing science software not based on C (e.g. C#, C++, Objective-C), serving as a scripting language for web applications such as those from Google, Yahoo, CERN (European Organisation for Nuclear Research) and NASA (National Aeronautics and Space Administration) [16]. The high-level and high-performance Python language can be installed in many operating systems supporting multiple programming paradigms (e.g. object-oriented, imperative and procedural styles). It is more effective than conventional languages (such as C and Java) in string manipulation, searching and cloud computing with lower memory consumption than Java, C and C++ [16]. Here, we use Python to develop a web-based open source software (SDMdata) for automatically or semi-automatically assisting in identifying and correcting errors from GBIF. The SDMdata web interface allows the process to be performed on any platform with a JavaScript-capable Internet browser. The output of the software is a file in CSV (comma separated values) format that can be widely used in other applications.

## SDMdata

**Fig 1:**
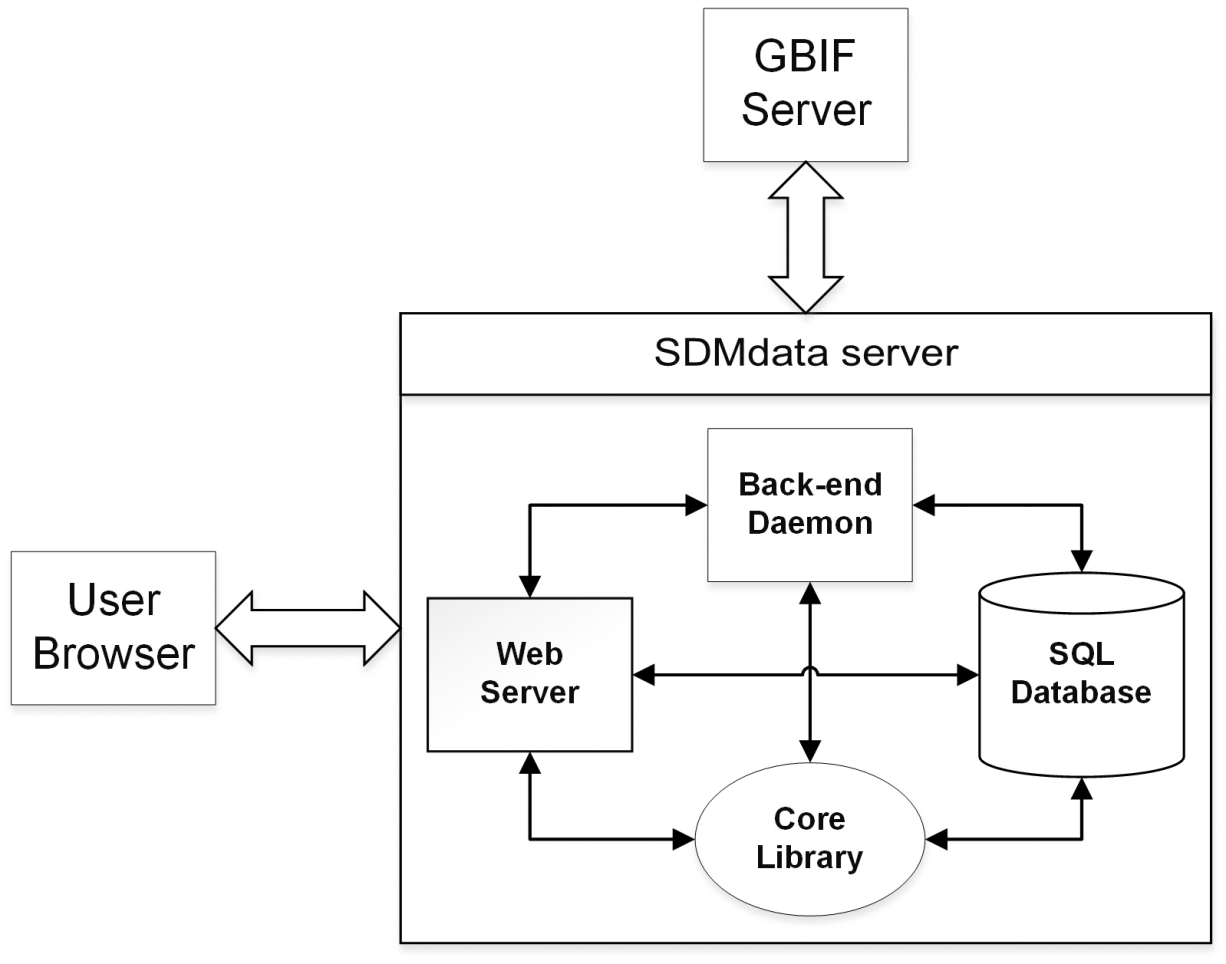
Structure of SDMdata.

SDMdata is designed for researchers to collect occurrence data from GBIF. After users import a file containing a list of species names, SDMdata will check the accuracy of species names against the GBIF database. The program will only proceed to collect species occurrence records if all of the species names are correct. After the collection job is complete, users can choose to do a check on the records to determine whether the coordinates have potential errors. See Fig 1 for whole system structures. SDMdata has some advantages over other software packages including: 1) SDMdata is based on Python, and thus has all the advantages of Python including high-performance and lower memory consumption. 2) The basic workflow of SDMdata is very easy, similar to the online submission system of academic periodicals, familiar to researchers. 3) SDMdata is web-based, meaning that users can access SDMdata from a web browser on any computer without any additional setup. 4) SDMdata has a user-friendly interface such that it can be used on many platforms (different operating systems) and many devices (e.g. desktop computer, tablet computer, or even a mobile phone). 5) SDMdata is released under an AGPL (GNU Affero General Public License) license. It is open source meaning that it is not only free to use, but its full source code is also freely accessible.

## The Basic Workflow of SDMdata

**Fig 2:**
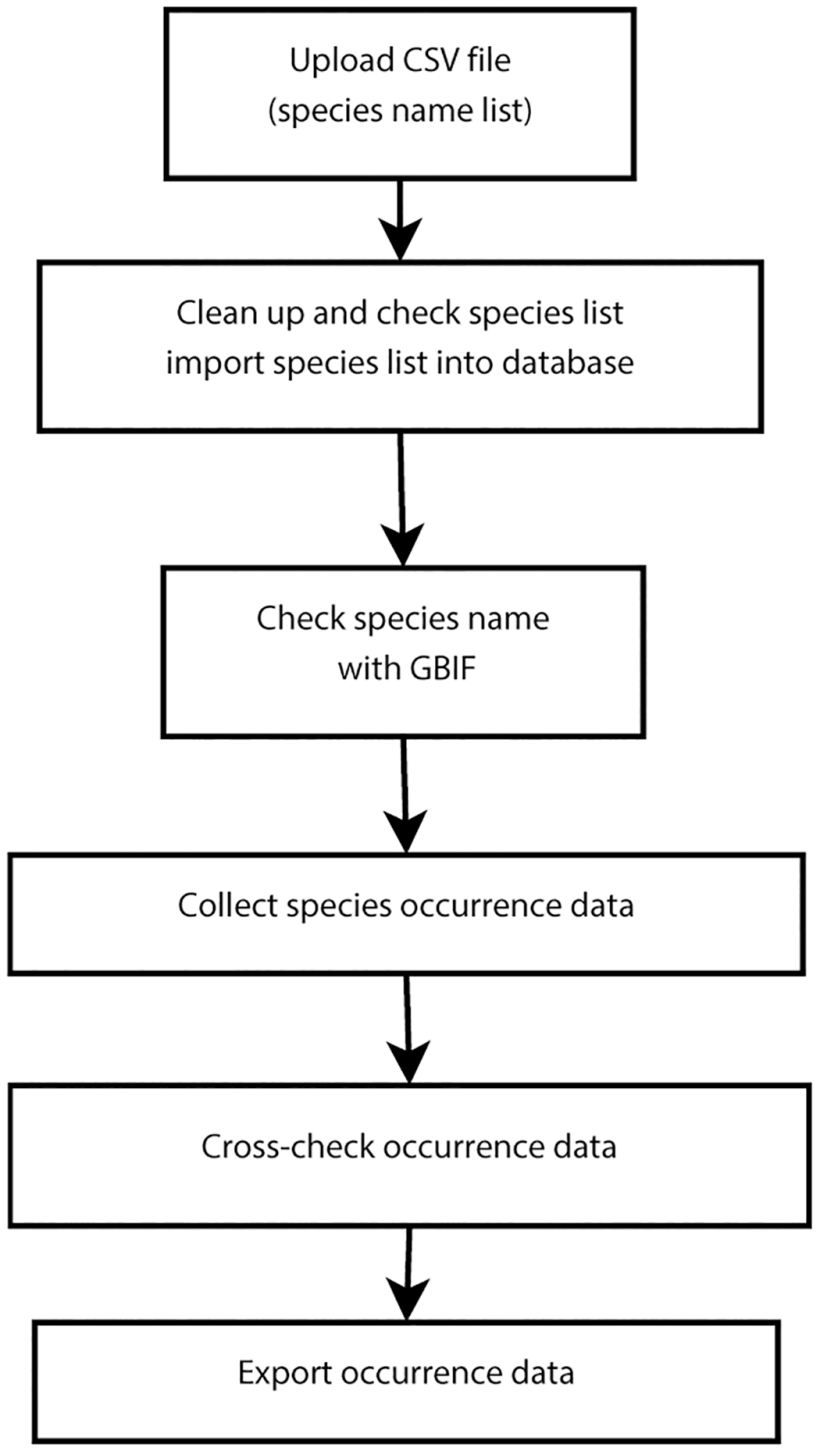
Workflow of SDMdata.

SDMdata has six basic steps within its workflow: 1) uploading a CSV file, 2) cleaning up, checking the species list, and importing the species list into the database, 3) checking the species name with GBIF, 4) collecting species occurrence data, 5) cross-checking occurrence data, and 6) exporting occurrence data (Fig 2). Steps 3–5 are somewhat complicated, and are described below in detail.

### Checking Species Names with GBIF

**Fig 3:**
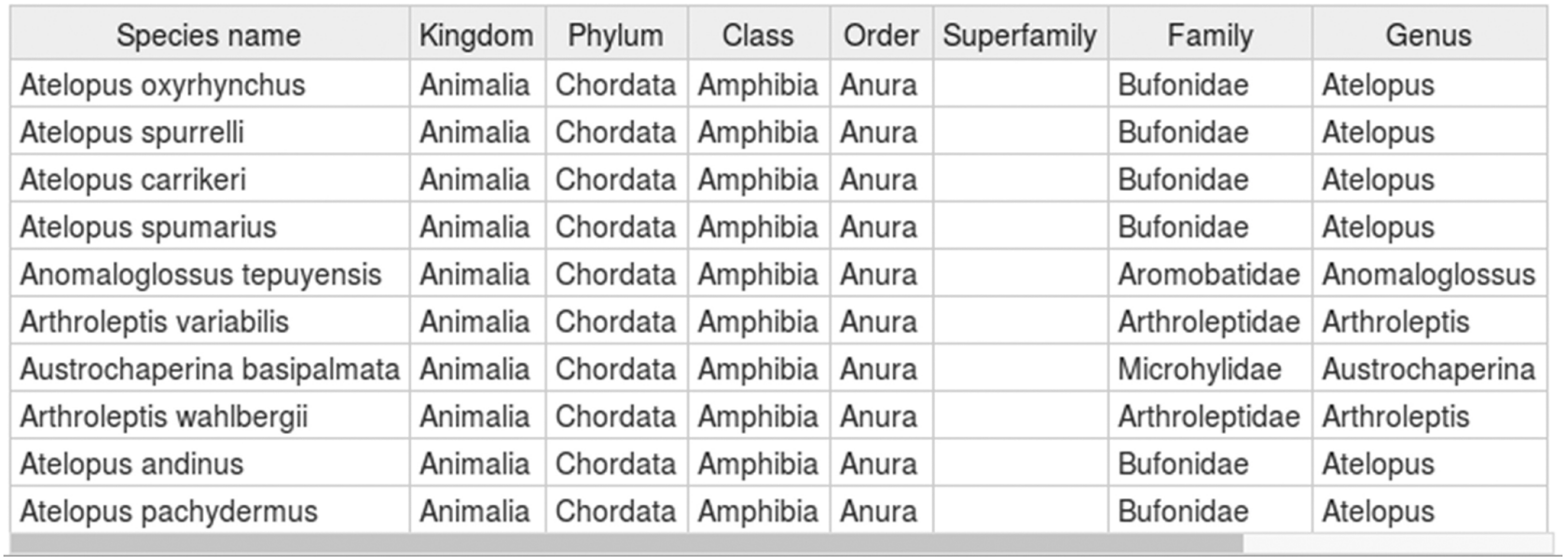
Species names with complete taxonomy information from GBIF.

Inputting species names is the first critical step to obtain species distribution information from species-occurrence databases. However, most ecologists and biogeographers have not completely mastered taxonomic knowledge, especially when many species are needed, and there may be some errors in species names (e.g. formatting errors or misspellings), which leads to problems in obtaining species information. SDMdata provides a function to check and extend species name information by using GBIF’s backbone taxonomy as the backend database (S1 Appendix). GBIF’s backbone taxonomy, often called the Nub taxonomy, is the largest species taxonomy database with a single synthetic management classification with the goal of covering all names in GBIF. It checks species names first, before the software collects the occurrence record, with two steps: 1) It checks whether users inputted incorrect species names (e.g. wrong spelling, changed species name). This is a common problem when researchers are dealing with tens of thousands of species. 2) It enhances species names with complete taxonomic information (including kingdom, phylum, class, order, family and genus). This is always useful in comparing the distribution of taxa (Fig 3).

### Collecting Species Occurrence Data

The core function of SDMdata is to fetch species occurrence points. This is the common function in similar software (e.g. rgbif, modestR), but SDMdata improves upon these with an independent daemon program. GBIF provides a web-based API to fetch occurrence points. It is easily impacted by the network and problems with DNS (Domain Name Service) or the network connection. This becomes important when data must be fetched for a long time without a break. All key functions of SDMdata (including checking species name, fetching occurrence records and crossing-checking) are written in an independent daemon program. All key functions have break-point memory protection. This means that it does not matter if the function is stopped at any time, and the software will simply finish the process upon restarting. This is useful when the user needs to stop the process or the network connection encounters a problem. All of the key functions are saved, meaning that an unexpected software or operating system crash or other fatal error will not prevent the software from completing its task.

### Cross-checking Occurrence Data

**Fig 4:**
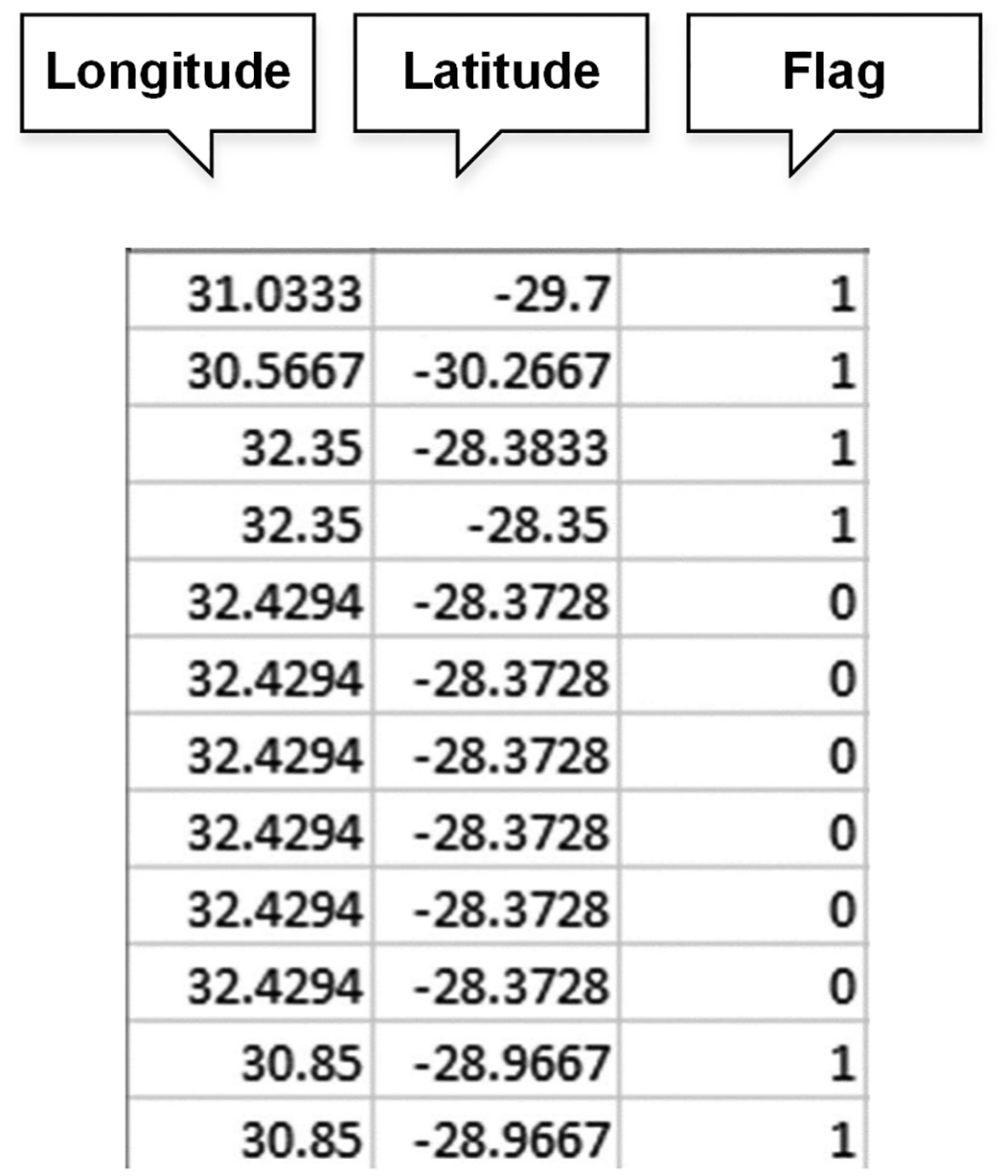
Result of cross-checking (0 indicates that geographic coordinates may be wrong, 1 indicates that geographic coordinates are correct).

Many occurrence records from observational data, survey data, as well as museum and herbarium data, have little geographic information or inaccurate geographic coordinates (latitude and longitude). Data errors are common and error rates can be up to 1~5% [17]. For data to meet the needs of users, error detection, validation and cleaning are essential parts of the data management process to improve data validation and correctness [18]. The two major errors in species occurrence points are spatial position (geographic coordinates) and identification error resulting for any number of reasons (e.g. GPS device errors, the exchange of longitude with latitude). We use cross-checking to find potential errors. There are many types of cross-checking, for example the quartiles method, jackknife method or autoselect best method [13]. SDMdata implements the most commonly used cross-check function for land-based species, which checks the geographic coordinates (latitude and longitude) with the geographic information of a specific country to identify potential errors [19]. Species occurrence records from GBIF have a country code recording the location of the sample. When the coordinate does not match the country code (the coordinate is not located in the country), this occurrence record may have an error (country border map is provided by GADM; http://www.gadm.org/). Note that a cross-check failure does not mean that this record is incorrect, but indicates to the researcher that this record is likely to be incorrect and requires closer inspection for errors. The accuracy of a country’s borders on the map, the accuracy of the GPS device, or other factors may mean that records located near the country’s border appear to be outside of the country. Our software will flag species occurrence records with problem for further checking (Fig 4, S2 Appendix).

## Technical Implementation

SDMdata is written in Python language with the geospatial process portion using GDAL library (http://www.gdal.org/) and the web server portion using the flask framework (http://flask.pocoo.org/). GDAL is widely used and the most important open source geospatial process library. SDMdata is currently available at <http://www.sdmserialsoftware.org/sdmdata/>.

## Supporting Information

S1 Appendix Workflow of uploading, importing and checking species names. (DOC)

S2 Appendix Workflow of fetching occurrence and cross-checking occurrence process. (DOC)
