# Supplementary material for: SDMdata: A Web-Based Software Tool for Collecting Species Occurrence Records
Source: PLoS One. 2015 Jun 1;10(6):e0128295. doi: 10.1371/journal.pone.0128295 (PMC4452258; doi:10.1371/journal.pone.0128295)
Supplement: S1 Appendix — (DOC) [file pone.0128295.s001.doc]

1. **Upload species name CSV file**


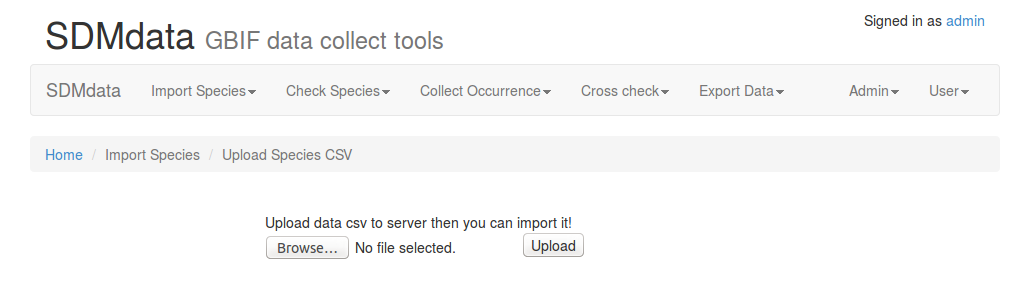


**(2) Import species name into software**


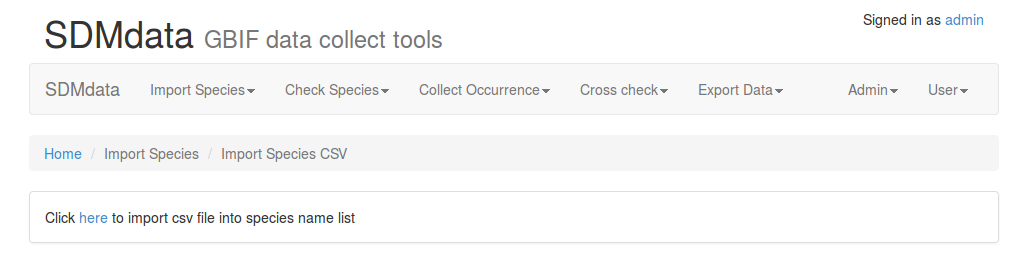


**(3) View imported species name (optional)**


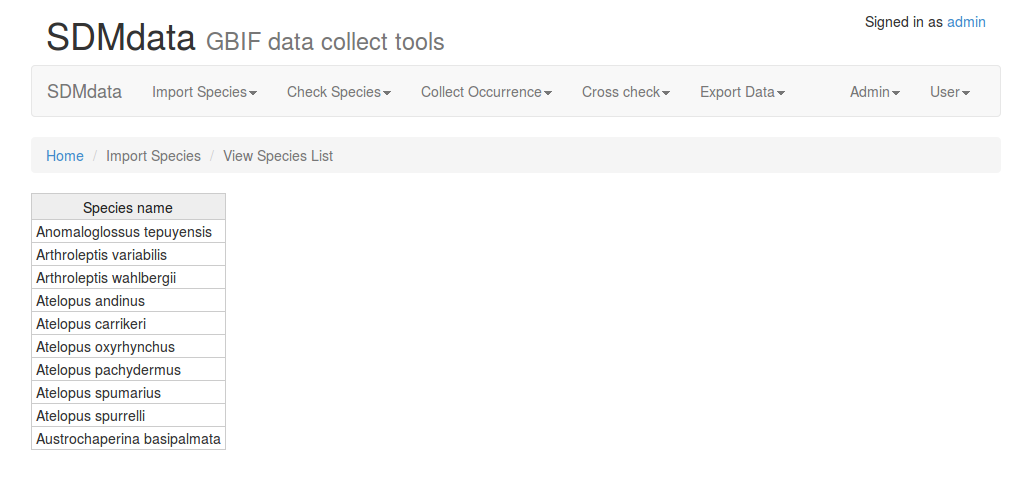


**(4) Check species name with GBIF**


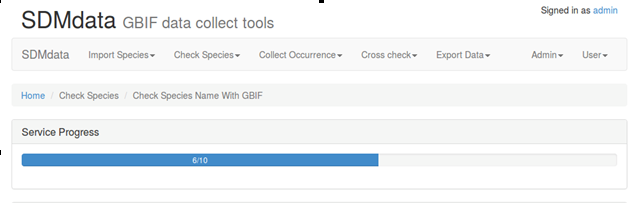


**(5) View checked species name (optional)**


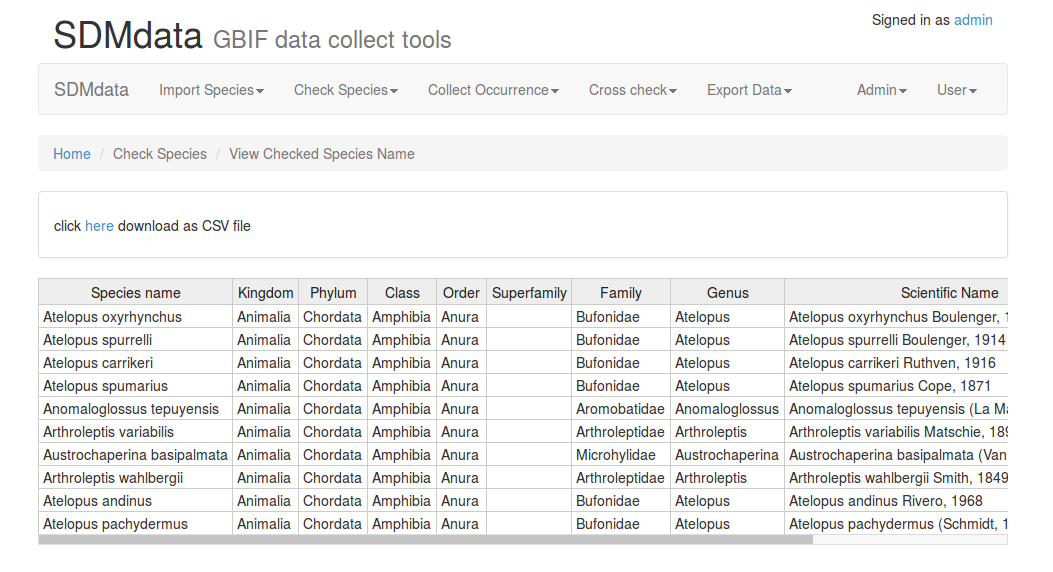


**(6) View error species name (optional)**


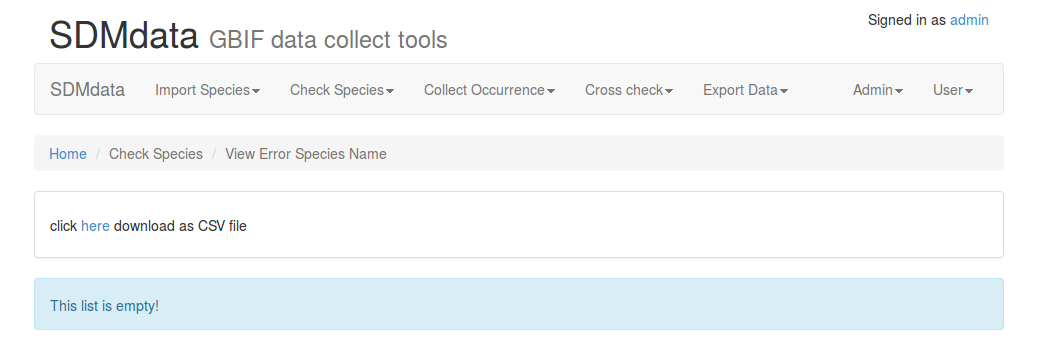


**Fig S1 Workflow of uploading, importing and checking species names**
