# Supplementary material for: SDMdata: A Web-Based Software Tool for Collecting Species Occurrence Records
Source: PLoS One. 2015 Jun 1;10(6):e0128295. doi: 10.1371/journal.pone.0128295 (PMC4452258; doi:10.1371/journal.pone.0128295)
Supplement: S2 Appendix — (DOC) [file pone.0128295.s002.doc]

1. **Fetching occurrence record**

**
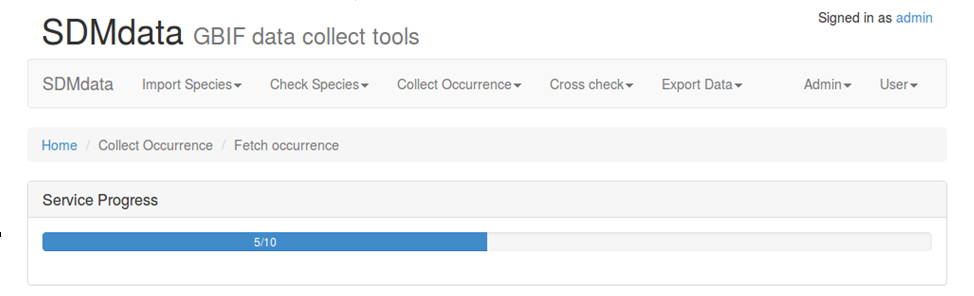
**

1. **Viewing species with no occurrence record**


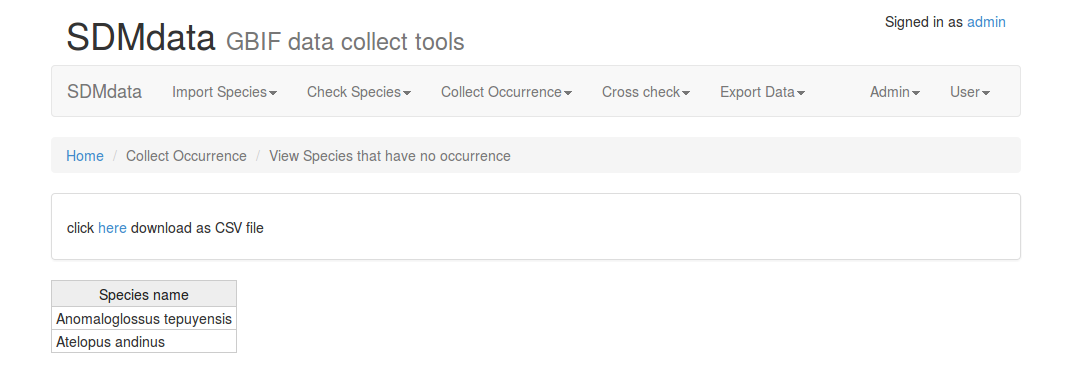


**(3)Viewing species with un-coordinate occurrence record**

**
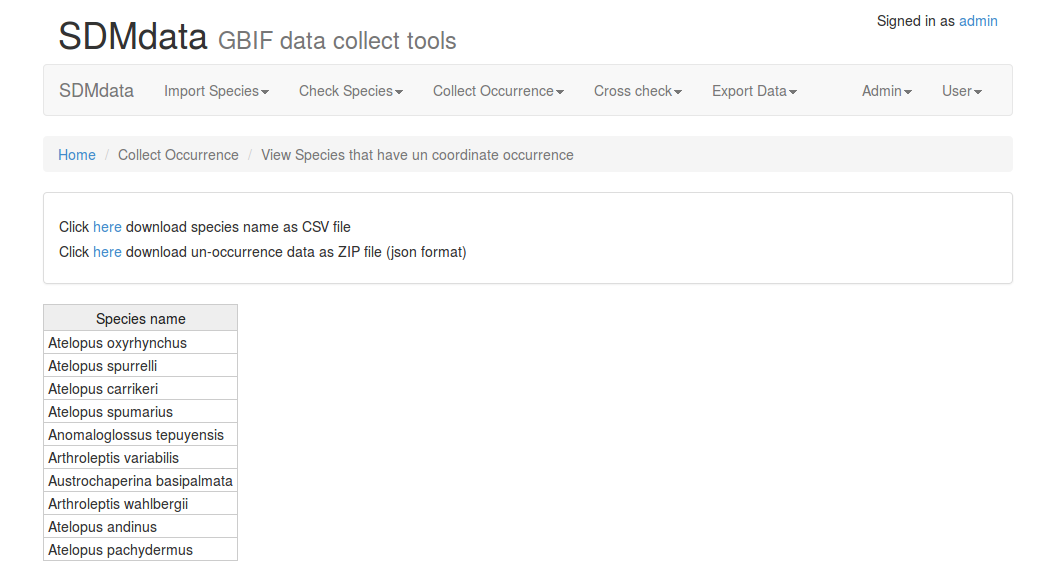
**

**(4) Crossing check the occurrence progress**


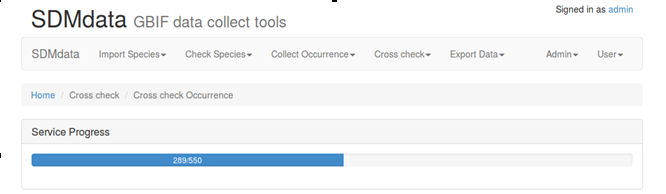


**(5) Viewing the status of crossing check**


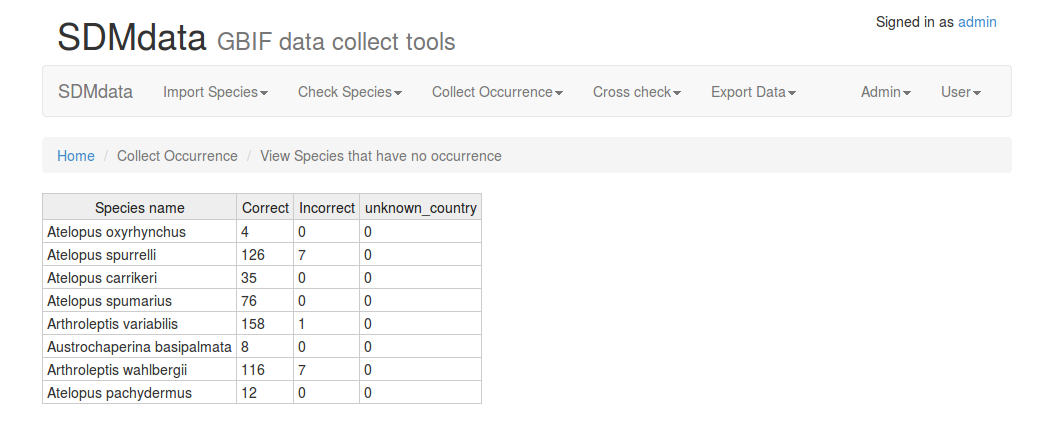


**(6) Viewing the export data**


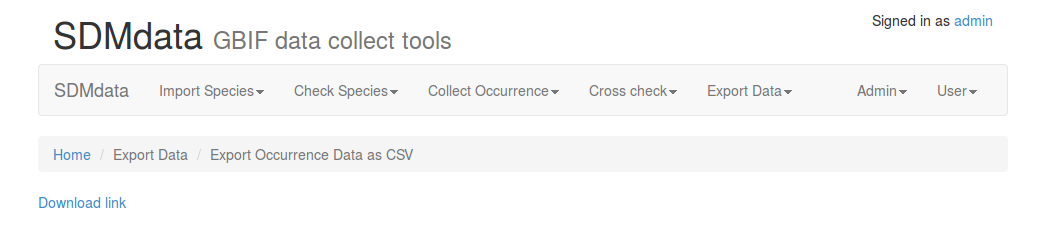


**Fig S2 Workflow of fetching occurrence and cross-check occurrence process**
